# Plant protein, fibre and physical activity solutions to address poor appetite and prevent undernutrition in older adults: study protocol for the APPETITE randomised controlled trial

**DOI:** 10.1017/S0007114524002125

**Published:** 2024-09-28

**Authors:** Katy M. Horner, Brian Mullen, Anna Quinn, Pia Scheufele, Susanne Gola, Federica Gonnelli, Matteo Bozzato, Jedd Pratt, Wiktoria Sala, Sinead Mullin, Laura Kirwan, Dominique Dardevet, Christelle Guillet, Giuseppe De Vito, Marjolein Visser, Dorothee Volkert, Clare A. Corish

**Affiliations:** 1 School of Public Health, Physiotherapy and Sport Science, University College Dublin, Dublin 4, Republic of Ireland; 2 Institute for Food and Health, University College Dublin, Dublin 4, Republic of Ireland; 3 Institute for Sport and Health, University College Dublin, Dublin 4, Republic of Ireland; 4 Institute for Biomedicine of Aging, Friedrich-Alexander-Universität Erlangen-Nürnberg, Nuremberg, Germany; 5 Fraunhofer Institute for Process Engineering and Packaging, Freising, Germany; 6 Department of Biomedical Sciences, University of Padova, Padova, Italy; 7 Department of Sport and Exercise Sciences, Manchester Metropolitan University Institute of Sport, Manchester, UK; 8 Université Clermont Auvergne, INRAE, UNH, Clermont-Ferrand, France; 9 Department of Health Sciences, Faculty of Science, and the Amsterdam Public Health Research Institute, Vrije Universiteit Amsterdam, Amsterdam, The Netherlands

**Keywords:** Ageing, Appetite, Exercise, Fibre, Hunger, Plant protein, Supplementation

## Abstract

Reduced appetite with ageing is a key factor that may increase risk of undernutrition. The objective of this study is to determine the impact of innovative plant protein fibre (PPF) products within a personalised optimised diet (PD), a physical activity (PA) programme, and their combination on appetite, and other nutritional, functional and clinical outcomes in community-dwelling older adults in a multi-country randomised controlled intervention trial. One hundred and eighty community-dwelling adults (approximately sixty per trial centre in Germany, Ireland and Italy) aged 65 years and over will be recruited to participate in a 12-week, parallel-group, controlled trial. Participants will be randomised into one of four groups: 1, PD (incorporating two PPF products): 2, PA; 3, PD + PA; and 4, no intervention (control). The primary outcome is appetite measured by visual analogue scales and energy intake from an *ad libitum* test meal. Secondary outcomes include fasting and postprandial appetite-related gut hormones, Simplified Nutritional Appetite Questionnaire score, body composition, cardiorespiratory fitness, muscle strength, physical function and PA. In addition, self-efficacy, cognitive status, dietary restraint, depressive symptoms and compliance and acceptability of the intervention will be assessed. Metabolomic profiles, RMR, muscle motor unit properties and gut microbiome will also be assessed to explore potential underlying mechanisms. This multi-centre randomised controlled trial will advance knowledge on how PD (incorporating PPF products), PA and their combination influence appetite, nutritional status and related health outcomes in community-dwelling older adults and contribute to the prevention of undernutrition. **Trial registration:** Clinical Trials.gov Registry NCT05608707 (registered on 2 November 2022). **Protocol Version:** NCT05608707 Version 4 (registered on 29 September 2023).

Older adults are at high risk of poor appetite and undernutrition, which can lead to frailty, sarcopenia and poor health outcomes^(1–7)^. Although definitions of appetite vary^(8)^, reduced appetite and food intake occurs even in healthy older adults and manifests alongside reductions in muscle mass^(3,6,9,10)^. A meta-analysis of fifty-nine studies comparing healthy older with younger adults reported hunger to be 25 % (fasting) to 39 % (postprandial) lower and energy intake to be 16–20 % lower in the older adults^(10)^.

Multiple environmental, psychological and physiological factors are implicated in appetite deterioration in older adults. For example, social isolation, depression, loss of pleasure in food and taste decline all contribute to reduced appetite^(11–13)^. Physiological factors include altered gut and other peripheral hormones related to appetite, reduced gut motility, inflammation and changes in the central control of food intake^(11,14)^. Reduced fat-free mass and resting energy expenditure and an altered gut microbiome have also been implicated as determinants of reduced appetite in older adults^(12,15,16)^, although evidence of causal associations is currently limited. Multicomponent intervention strategies are therefore needed to address appetite decline and prevent undernutrition^(2,12)^.

The need for interventions to address reduced appetite in older adults, or to increase dietary intake despite a reduced appetite, has been clearly recognised and should be a key target for reducing nutrient deficiencies and risk of undernutrition^(15,17)^. However, current pharmacological interventions have limited utility in clinical practice^(15)^. Appropriate nutrition and exercise are considered the optimal strategies to limit declines in muscle mass and function with ageing^(18,19)^. Adequate protein and dietary fibre intakes are essential for healthy ageing. Adequate protein intake is important to reduce age-related declines in muscle mass, strength and function^(20,21)^. Adequate fibre intake is associated with normal bowel function, reduced intestinal transit times and reduced risk of several non-communicable diseases, among other health benefits^(22–24)^. For older adults, daily consumption of at least 1·0–1·2 g/kg body weight of protein^(18,19)^ and 25–30 g of fibre^(24,25)^ is recommended. However, intakes of both nutrients are often sub-optimal in European older adults^(23,26–31)^, including in those with poor appetite^(27)^. In a cross-sectional analysis of over 2500 community-dwelling older adults, those with a poor appetite were found to have a lower intake of protein and dietary fibre compared with those with good appetite, after adjustment for energy intake and other potential confounders^(27)^.

With regard to effects on appetite and energy intake, a systematic review of acute and longitudinal studies found protein supplementation (through supplements or whole foods) has no negative impact on overall energy intake in older adults^(32)^. Similarly, a systematic review of thirty-eight different fibre sources found most isolated fibres did not acutely suppress appetite or energy intake in the general population and a recent 10-week intervention in older adults demonstrated pea hull fibre (10 g/d) supplementation did not suppress appetite^(33,34)^. Interestingly, older adults with poor appetite also appear to have a preference towards non-dairy and high-fibre foods^(35)^. Therefore, addition of plant protein and fibre to the habitual diet appears a promising strategy to improve intake of these nutrients and enhance related health outcomes. Both nutrients may impact several mechanisms implicated in appetite decline with ageing such as alterations in gut motility, inflammation, fat-free mass and the gut microbiome and metabolite profiles^(11,14,16,24,36–41)^. However, further longitudinal studies examining the effect of protein and fibre on appetite and energy intake in older adults are needed.

There is debate surrounding optimal protein sources to support healthy ageing. Although plant proteins are generally characterised by a lower content of essential amino acids, a meta-analysis of nine studies (including three studies in adults aged over 50 years) found longer-term supplementation with animal or plant protein combined with resistance exercise produced similar effects on lean mass and strength^(42)^. Furthermore, evidence from cell and animal models suggests that insoluble fibre may enhance *de novo* amino acid biosynthesis via alterations in the gut microbiome^(41,43)^, which would support combining plant protein with fibre supplementation. Plant proteins are also well accepted by older adults and more environmentally sustainable^(44,45)^.

Consensus guidelines on nutrition for older adults highlight the importance of individualised nutrition interventions and recommend including additional non-dietary factors such as physical activity (PA) or social aspects^(2)^. In healthy older adults, most benefits of protein supplementation are only observed when combined with exercise^(46)^. There is initial evidence that whey protein supplementation combined with exercise can increase energy intake in older adults^(36)^. Elsewhere, it has been hypothesised that increasing PA may benefit appetite and energy intake, although the evidence base to support this is currently limited^(47,48)^.

The innovative plAnt Protein fibre and Physical activity solutions to address poor appEtite and prevenT undernutrITion in oldEr adults (APPETITE) randomised controlled trial (RCT) aims to determine the impact of a personalised optimised diet (including innovative plant protein fibre (PPF) products), a PA programme, and their combination on appetite and other nutritional, functional, metabolic and clinical outcomes in community-dwelling older adults. The multi-country trial is part of the European APPETITE project, funded under the Horizon Joint Programming Initiative ‘A Healthy Diet for a Healthy Life’^(49)^.

## Methods and analysis

The protocol is written in accordance with the Standard Protocol Items: Recommendations for Interventional Trials (SPIRIT) guidelines^(50)^. A summary checklist is reported in Appendix 1(online Supplemental Appendix 1). The composition, roles and responsibilities of the coordinating centre, individuals overseeing the trial and external advisory board are listed in Appendix 2 (online Supplemental Appendix 2).

### Study design

The trial is a multi-centre RCT aiming to recruit 180 community-dwelling older adults (about 60 per centre) in Nuremberg – Germany, Dublin – Ireland and Padua – Italy, all urban settings. Employing a 2 × 2 factorial parallel-group design, outcome measures will be assessed pre and post a 12-week intervention. Participants will be randomly allocated (1:1:1:1) by study centre and sex to one of four groups:

1. Personalised diet (PD): daily consumption of two PPF products and dietary counselling over a 12-week period;
2. PA: group exercise classes twice per week and home-based walking over a 12-week period;
3. PD and PA: both interventions described above combined;
4. Control: no intervention, usual diet and PA.

**Fig. 1.**
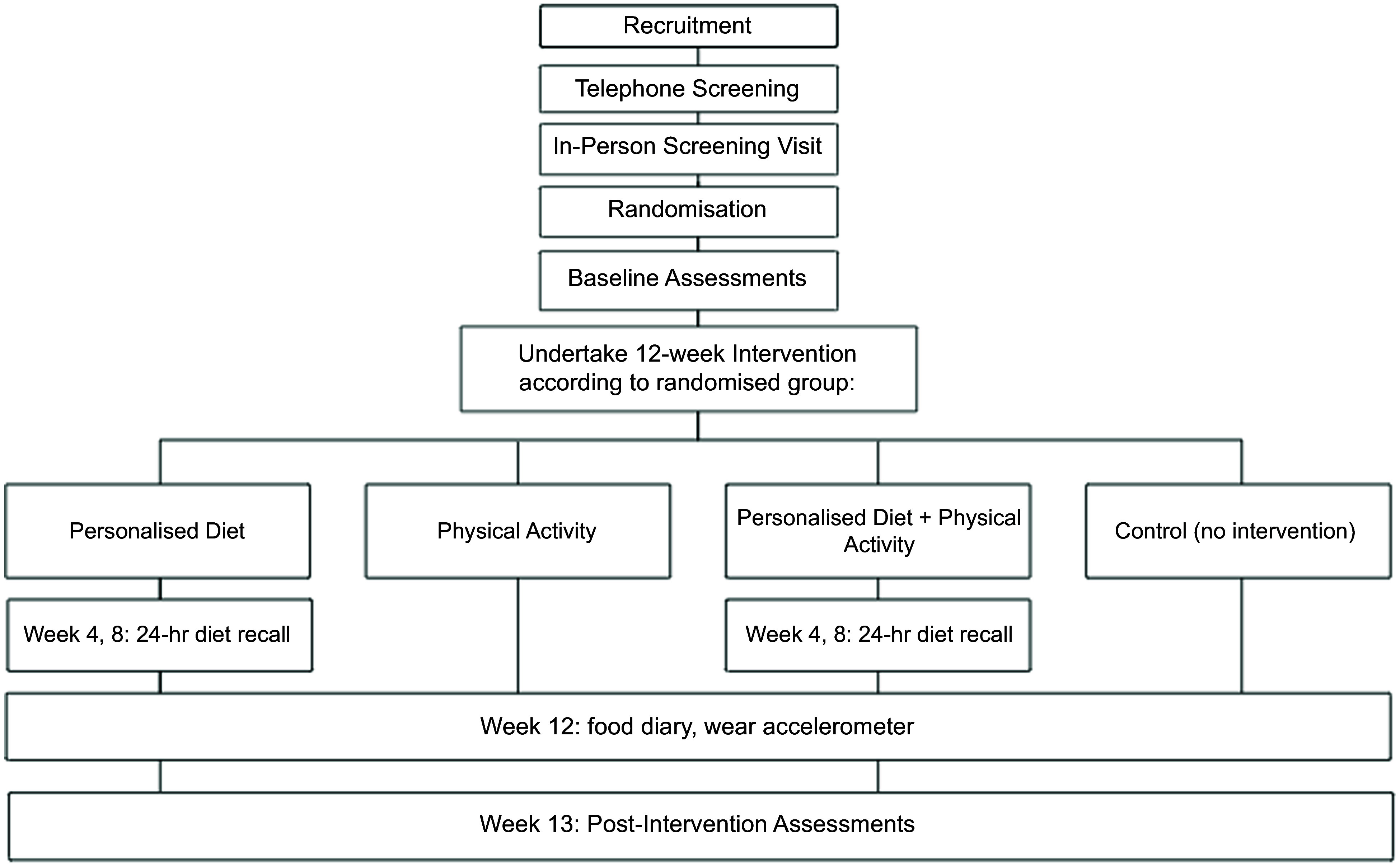
Overview of timing of screening, intervention and assessments. Primary outcomes are assessed at baseline and week 13. Created in Biorender.com

An overview of the timeline for participants is shown in Fig. 1.

Screening (telephone followed by in-person) and baseline (pre-intervention) assessments will be completed within 6 weeks prior to commencing the trial. The intervention duration lasts 12 weeks. Post-intervention testing will be performed in the week following the intervention. In groups involving PD, participants will be required to consume PPF supplements and in groups involving PA to complete home-based walking up to and including the day prior to returning for the final outcome assessment, respectively.

#### Sample size

Sample size calculations were conducted in G * Power (version 3.1). A 120 kcal (500 kJ) difference in *ad libitum* test meal energy intake and a 10 mm change in visual analogue scale (VAS) appetite ratings have previously been considered physiologically relevant differences^(8,51)^. To allow for 20–25 % dropout, a sample size of 180 participants (45 per group) is sufficient to detect these differences between groups, allowing for 10 % variance on a within participant sd of change in *ad libitum* test meal energy intake of 143 kcal ^(36,52)^, and for VAS of about 13 mm, observed in our unpublished data, similar to others^(36)^, at 80 % power and significance at *P* < 0·05.

#### Recruitment and eligibility of study participants

Multiple recruitment strategies will be employed including flyers, social media and newspaper advertisements. If recruitment does not meet targets in one centre, this may be compensated for by additional recruitment at another centre. Interested individuals will be provided with a participant information sheet, and if still interested and potentially eligible will complete telephone screening with a trained researcher. Persons who are still eligible after telephone screening will subsequently be invited to complete an in-person screening at the study centre. Criteria to determine participant eligibility are shown in Table 1. Medical conditions and medications for exclusion are listed in Appendix 3 (online Supplemental Appendix 3). Due to initially very slow recruitment, age was lowered from originally >/= 70 years, a Simplified Nutritional Appetite Questionnaire (SNAQ) score </=14 removed (both April 2023) and BMI widened from 22 to 27 kg/m^2^ (August 2023). Most previous studies examining appetite in older groups and showing reduced appetite and energy intake compared with younger groups involved older adults aged 60–65 years and older^(10)^.

**Table 1.** APPETITE trial inclusion and exclusion criteria

Inclusion criteria	Exclusion criteria
• >/=65 years of age • Community-dwelling • BMI 20–30 kg/m^2^ • Inactive (<150 min moderate or vigorous activity/week) • Have proof of COVID-19 vaccination	• MMSE </= 24 • CES-D > 16 • Weight loss (intentional or unintentional) >5 % within last 3 months (participant self-report) • Heavy smoker (>10 cigarettes per d or vape equivalent) • Medical condition or medication known to impact on appetite or energy intake • Other medical condition that may impact on ability to participate in study or study outcomes • Planned relocation within the next 6 months • Inability to travel to study centre/PA location • Currently participating in another intervention study • Currently participating in regular resistance training > once per week • Inability to participate in physical activity • Unable to walk across a room • Allergic and/or unwilling to consume study test food • Loss of taste or smell associated with COVID-19 • Unwilling to accept randomisation to any study group

MMSE, Mini Mental State Examination; CES-D, Centre of Epidemiology Study – Depression (CES-D) questionnaire; PA, physical activity.

### Randomisation, blinding and study allocation

Participants will be randomised upon meeting all eligibility criteria on completion of in-person screening and will be informed of their allocated group after baseline data have been collected. Randomisation will be stratified by study centre and sex. If a participant is identified as ineligible after randomisation and prior to baseline assessment due to error in screening^(53)^, a participant of the same sex will be randomised to the group the ineligible participant was allocated to. If members of the same household are eligible, they will be stratified to the same group. Randomisation will be centralised and conducted by an independent researcher, not connected to the project, using the QMINIM platform (Telethon Kids Institute, Australia). For the duration of the intervention and post-intervention assessments, both participants and researchers are unblinded to intervention allocation.

### Interventions

#### Personalised diet

The PD intervention involves individual dietary counselling by a dietitian/trained nutritionist/research scientist and daily consumption of PPF products (detailed description below). The intervention aims at achieving the following predefined nutritional targets:

1. Daily consumption of 2 × 25 g PPF powders added to food/drinks.
2. Daily energy intake of 25–30 kcal/kg body weight^(54)^.
3. Daily protein intake of 1–1.2 g/kg body weight from food and PPF^(55)^.

These nutritional goals are based on previous research in older adults^(54,55)^. As recommended by expert consensus statements^(2)^, participants will be regularly monitored and nutritional targets will be adjusted according to each participant’s current nutritional status and co-morbidities requiring specific dietary strategies. One personalised dietary advice session (about 45 min) will be delivered in person at the start of the intervention, and two 35–45-min sessions via telephone (weeks 4 and 8). In addition, fortnightly compliance telephone calls will be conducted by a trained member of the research team. The personalised dietary advice will be based on the following assessments: (i) 3-d food diary (week before starting intervention, see below), (ii) nutritional assessment at baseline and (iii) two single 24-h dietary recalls via telephone (weeks 4 and 8).

As part of the nutritional counselling, participants will be provided with a ‘Hints and Tips’ booklet with suggestions, including recipes, on how to incorporate the PPF powders into their everyday foods/meals/snacks/beverages, preferably in meals, snacks and/or beverages that contain 6–7 g protein from other sources (e.g. 20–25 g meat, 40 g fish, one egg, glass of milk, 125 ml yogurt, 25 g cheese, 2 thick slices bread, 25 g almonds or Brazil nuts), to enhance circulating methionine, as methionine appearance was found to be limited following PPF consumption^(56)^. As a priority, participants will be instructed to include a full 25 g sachet of PPF powder at two meals where protein intake is identified as being low (e.g. breakfast and lunch), to optimise protein distribution^(20)^. Participants will only be advised to spread the two sachets across multiple eating occasions if they report significant difficulty with consuming a full PPF sachet at one meal.

Where needed, participants will also be provided with country-specific resources that provide simple advice on how to address poor appetite and meet energy requirements to support the participant achieving a daily dietary intake of 25–30 kcal/kg and 1–1·2 g protein/kg body weight., for example, Nutrition supports – HSE.ie^(57)^.

#### Description of plant protein fibre products

Initially six varieties of PPF blends from different plant sources were developed by Fraunhofer Institute for Process Engineering and Packaging (IVV) (Freising, Germany) as part of the APPETITE project^(49)^. The PPF blends were designed to provide equivalent leucine and closely matched branched chain and indispensable amino acids and align with optimal values described for older adults to promote muscle mass and strength^(58)^. Their acceptability in a food matrix (natural yogurt) and sensory properties were subsequently tested by older adults (> 65 years, *n* 25) at two trial centres (FAU, Germany and UCD, Ireland). The three blends with the greatest acceptability had mean (sd) Likert scale (0–9 point) scores of 5 (2), corresponding to ‘neither like nor dislike’, similar to acceptability of oral nutritional supplements in older adults reported in previous studies^(59)^.

These three PPF blends were subsequently investigated for circulating plasma amino acid appearance following ingestion of the products in older adults, with results reported elsewhere, including quantitatively measured amino acid composition of the blends^(56)^. Based on these findings, two blends (referred to as PPF A and PPF B) were selected for the RCT. PPF A consists of 54 % pea protein isolate, 17 % oat protein concentrate, 9 % almond protein concentrate and 20 % vegetable pea fibre, with a 25 g sachet providing 15·3 g protein and 3·6 g fibre. PPF B consists of 36 % pea protein isolate, 26 % soya protein isolate, 18 % rice protein isolate and 20 % vegetable pea fibre, with a 25 g sachet providing 17·1 g protein and 3·6 g fibre. The ingredients used for the PPF formulation are commercial protein ingredients resulting from dry and wet fractionation of plant raw materials. The fibre ingredient is a commercial product derived from pea kernels after protein extraction. The protein contents of the ingredients and the nutritional composition of the PPF are provided in more detail in the previous publication of de Marco Castro *et al.* ^(56)^ and in Appendix 4. Participants will be given the same quantity of each PPF and advised to consume one sachet of each type per d.

### Physical activity

The PA intervention involves group exercise classes in a supervised setting twice per week (with at least 48 h between training sessions) and home-based walking three times per week, following a similar format to previous multi-centre trials in community-dwelling older adults ^(54,60)^.

The group exercise class will begin with a warm-up including training on walking dynamics. This will be followed by approximately 3–4 balance exercises. Resistance exercise will then be conducted in line with guidelines for resistance exercise in older adults^(61)^. Upper and lower body exercises will be completed using adjustable weights across centres including the following exercises: wide leg squat, standing leg curl, hip flexion, knee extension, seated row, bicep curls, seated shoulder press, along with push-ups (against a wall). The exercise intensity will progressively increase so that all participants should be achieving an intensity of 15 to 16 on the Borg 6–20 rate of perceived exertion (RPE) scale^(62)^. Weights will be increased individually so that this intensity is maintained throughout the remainder of the programme. For each strength exercise, participants will be instructed to perform 8–12 repetitions (one set), rest for approximately 1 min and then perform subsequent sets. The sets and repetitions prescribed will progressively increase over the 12-week programme. For example, in week 1, participants will perform eight repetitions (one set) per exercise, rest for approximately 1 min and then perform a second set. As the participants progress, repetitions will increase from 8 to 10 to 12 and sets will increase from 2 to 3 sets per exercise.

For home-based walking, the goal is 60 min (3 × 20 min) of walking per week during the first 3 weeks, 90 min (3 × 30 min) per week for weeks 4–6 and finally 120 min (3 × 40 min) per week for weeks 7–12. The walking goals may be accumulated over the day until participants are able to achieve the continuous duration. Walking pace should be at a moderate intensity defined as an RPE of 13 on the Borg 6–20 scale^(62)^. For individuals who are very deconditioned, it is acceptable to exercise at an RPE < 11 in the initial 3 weeks until they become more comfortable with the exercise protocol.

#### Personalised diet and physical activity

Participants in the personalised diet and physical activity (PD + PA) group will undertake both PD and PA interventions combined. Participants will follow identical procedures to those described in the PD and PA interventions above.

#### Control

The control group will receive no intervention, and participants are instructed to maintain their usual diet and PA which is considered standard care for older adults with poor appetite across study centres. Participants will be provided with tailored nutritional and lifestyle information following study completion.

### Maximising and monitoring compliance with personalised diet and physical activity interventions

Adequate weekly compliance in the PD group is defined as self-reported consumption of at least ten of a possible fourteen PPF powder sachets in that week, similar to previous supplementation studies^(63)^. Participants will be asked to complete a daily log, ticking a box to indicate that they have consumed each powder sachet. In addition, participants will be asked to keep all empty sachets in a container provided by the research team at the baseline test visit, as an additional objective measure of compliance. This must be returned at the end of the study. Overall compliance is defined as adequate compliance for at least 9 weeks during the 12-week intervention (75 %).

During the fortnightly compliance telephone calls in the PD and PD + PA groups, compliance with the intervention will be encouraged and problems troubleshooted. For participants in the PD + PA group, this may instead be conducted in person before/after an exercise class, and for participants in the PA group it will be conducted in person before/after an exercise class to reduce participant burden.

Adequate compliance in PA groups is defined as completing a minimum of 75 % of group exercise sessions (i.e. a minimum of eighteen out of twenty-four sessions). Attendances will be recorded by the instructor at all supervised sessions. Home-based walking and all moderate to vigorous activity will be recorded in the compliance log. Compliance logs will be checked by trained researchers throughout the intervention at exercise sessions.

If participants do not adhere to the above interventions, they will be included in intention-to-treat analysis and excluded from per-protocol analysis.

### Data collection and study outcomes

Data will be collected on two separate test visits at baseline and two identical visits post-intervention (week 13) (Fig. 1). One visit consists primarily of assessment of physical characteristics and function, and the other visit consists of an appetite test meal challenge. Participants will be instructed to avoid strenuous exercise and alcohol in the 24 h before testing on both occasions. Participants will also be instructed to fast for at least 10 h overnight, prior to arrival at the laboratory, and to consume nothing other than water during this time. A small glass of water is advised upon waking on the morning of testing. Participants will be instructed to follow their normal medication routine, and any smokers that are eligible will be instructed to avoid smoking/vaping on the morning of testing. A standardised snack will be provided to participants prior to physical function testing. For post-intervention testing, identical procedures will be followed. A summary of the data collection variables/measures and time points of collection is shown in online Supplemental Appendix 5.

### Primary outcomes

#### Subjective appetite and ad libitum test meal energy intake

The joint primary outcomes are (i) postprandial composite appetite score (area under the curve from 0 to 180 min (AUC_0–180 min_)) derived from three 100 mm VAS (hunger, fullness and desire to eat) and (ii) energy intake (kilocalories (kcal)) from an *ad libitum* lunch test meal. Appetite ratings will be assessed at the following time points: fasting before (*t* –10 min) and after a standardised breakfast (see below) (0 min, 30 min, 60 min, 120 min, 180 min and post-lunch). This time frame represents a typical inter-meal interval during which time an oscillating pattern of hunger and fullness would be expected^(64,65)^. Composite appetite score is calculated as follows: (VAS hunger + VAS fullness (100-rating) + VAS desire to eat)/3. On either end of the VAS 100 mm line are opposing terms (i.e. ‘not at all’ and ‘extremely’). The trapezoid method will be used for the calculation of AUC. The *ad libitum* test meal will be served at 180 min and energy intake will be assessed.

The joint primary outcomes were selected to include a subjective and an objective measure of appetite, reflecting different processes of appetite control^(64)^. VAS have a history of widespread use and acceptance across many countries and laboratories, and according to consensus recommendations, they are easily used and translated and appear to be a valid, sensitive and unbiased measurement tool of appetite^(8)^. VAS have been validated for assessment of appetite, including in older adults^(66–68)^.

#### Test meals

The fixed breakfast meal consists of white bread toasted (88 kcal), butter (36 kcal), jam (24 kcal), orange juice (82 kcal) and strawberry yogurt (111 kcal), providing approximately 340 kcal overall, 57 g carbohydrate (66 % energy), 8 g fat (22 % energy) and 11 g protein (12 % energy) across all centres.

The *ad libitum* lunch test meal consists of a homogenous pasta-bake meal, identified as being acceptable to older adults across all centres. The key ingredients are pasta, tomato-based pasta sauce, cheese and olive oil, providing approximately 1900 kcal, 17 % energy protein, 54 % energy carbohydrate, 28 % energy fat (maximum 1 % energy deviation between centres). The meal is freshly prepared and provided in a large serving dish with utensils and a bowl into which participants help themselves. Participants are instructed that they have 30 min to help themselves to and eat as much as they wish until they feel comfortably full. Water intake is recorded and the amount of food consumed is determined by weighing the meal before and after consumption. Energy intake is calculated using the manufacturers’ energy values.

### Secondary outcomes

#### Anthropometric measurements and body composition

Height, weight and seated maximum calf circumference of the dominant leg will be measured in duplicate using a stadiometer, digital scale and flexible measuring tape, respectively. Body resistance at 50 kHz will be measured by bioelectrical impedance analysis (BIA) at all sites as follows: Germany: (Impedimed Imp SFB7, Impedimed Limited, Queensland, Australia), Ireland: Bodystat QuadScan 4000, Bodystat Ltd, UK; Italy: BIA 101 BIVA® PRO, Akern Systems, Firenze, Italy) in a fasted state. Appendicular skeletal muscle mass, fat mass and fat-free mass will be estimated using validated equations in older adults^(69,70)^. Fat mass and fat-free mass will additionally be assessed by air displacement plethysmography (BodPod, Cosmed, Italy) at one site (Ireland), and fat mass, fat-free mass and appendicular skeletal muscle mass by dual-energy X-ray absorptiometry at one site (Italy).

#### Simplified Nutritional Appetite Questionnaire

Appetite will also be assessed via SNAQ^(71)^, a self-administered questionnaire adapted from the Appetite, Hunger and Sensory Perception questionnaire^(72)^. The four-item SNAQ includes questions on appetite, fullness, taste and frequency of eating and has been shown to predict weight loss in community-dwelling older adults^(57)^. It will be completed at baseline during in-person screening and post-intervention on the test day assessing physical characteristics and function.

#### Fasting appetite ratings

Fasting appetite ratings for hunger, fullness and desire to eat will be assessed using 100 mm VAS prior to consumption of the fixed breakfast meal.

#### Palatability of test meals

Palatability of the breakfast and lunch meals will be assessed using six 100 mm VAS. Questions include how pleasant was the meal, how satisfying was the meal, how tasty was the meal, how filling was the meal, how sweet was the meal and how savoury was the meal. Each end of the VAS is anchored with the words ‘not at all’ and ‘extremely’.

#### Dietary intake

Energy, protein and fibre intake will be assessed using a 3-d non-weighed food diary administered at baseline (pre-intervention) and in the final week of the intervention or control period. Participants will be instructed orally and in written form to record all items of food and beverages consumed and provide details of the brand, estimated quantity and cooking methods used. Energy and nutrient intake will be calculated using national nutrient databases ^(73,74)^.

#### Cardiorespiratory fitness, physical function and strength

Cardiorespiratory fitness will be estimated using a usual-paced 400 m walk test^(75)^. Physical function will be assessed by Short Physical Performance Battery (SPPB) combining the assessment of balance, gait speed and repeated chair stands tests^(76)^. Individual test scores and overall score will be calculated. Leg strength of the dominant leg will be assessed as the maximum value from three trials using a seated leg extension dynamometer to determine maximal voluntary isometric contraction of the knee extensors. Handgrip strength will be assessed as the maximum value from three trials with a Jamar hydraulic grip strength dynamometer, with the participant in a seated position and arm rested on the table at a 90-degree angle using the dominant hand, unless contraindicated.

#### Physical activity

PA and sedentary behaviour (including steps and METs, time spent walking, sedentary time and sit to stand transitions per d) will be assessed by ActivPAL accelerometer (activPAL3^TM^ micro, or activPAL4; PAL Technologies Ltd). The participant will be provided with the accelerometer to wear on the midline of the thigh for 7 d at baseline prior to commencing the intervention and during week 12 of the intervention or control period. Participants will also complete the Physical Activity Scale for the Elderly (PASE) questionnaire^(77)^. The PASE questionnaire is a brief and easily scored survey designed specifically to assess PA duration, frequency and exertion over a 7-d period in individuals 65 years and older. At post-intervention, participants will be instructed to include activities undertaken as part of the intervention in their response.

#### Resting energy expenditure

Resting energy expenditure (kcal/d) will be assessed following an overnight fast in participants at one site (Ireland) using indirect calorimetry (Q-NRG, Cosmed).

#### Cognitive status, dietary restraint and depressive symptoms

Cognitive status will be assessed by Mini Mental State Examination (MMSE)^(78)^. Cognitive dietary restraint will be assessed using the cognitive dietary restraint subscale of the Three-Factor Eating Questionnaire (TFEQ)^(79)^. The subscale consists of six ‘true/false’ and Likert scale-type questions relating to cognitive dietary restraint from the original fifty-item questionnaire. Depressive symptoms will be assessed by Centre of Epidemiology Study – Depression (CES-D) questionnaire^(80)^.

#### Quality of life

Subjective rating of perceived Quality of Life will be assessed using the EuroQol (European Quality Of Life) questionnaire (EQ-5D-5L)^(81)^. This consists of self-assessed rating of health under five dimensions (Mobility, Self-Care, Usual Activity, Pain/Discomfort, Anxiety/Depression) and perceived overall health on a VAS (0–100) anchored with opposing statements (0= the worst health you can imagine; 100= the best health you can imagine).

#### Blood biochemical measures

A fasting venous blood sample will be collected from participants at all sites prior to consumption of the fixed breakfast meal at baseline and post-intervention. Serum, lithium heparin and EDTA aprotinin tubes will be collected, processed according to standardised protocols and samples stored in aliquots at –80°C until analysis. Biomarkers related to appetite including ghrelin, GLP-1, PYY, glucose, insulin and leptin will be analysed centrally at one site (Ireland), except for plasma samples which will be transferred to National Research Institute for Agriculture, Food and the Environment (INRAE), France, for metabolomic analysis.

Postprandial samples in response to the fixed breakfast meal will also be collected at one site (Ireland) at baseline and post-intervention. Along with fasting measures, appetite-related gut hormones (ghrelin, glucagon-like peptide-1 (GLP-1) and peptide YY (PYY) will be assessed at 30, 60, 120 and 180 min post-breakfast until the *ad libitum* lunch test meal is served at 180 min and analysed in Ireland. In addition, metabolomic analysis will be conducted at INRAE, France, on postprandial samples collected at the same time points. At each site, all samples will be analysed together upon completion of the trial.

#### Gut microbiome diversity and gut metabolomic profiles

Gut microbiome diversity and metabolomic profiles will be assessed from a faecal sample and faecal water collected at baseline and post-intervention from participants at two sites (Ireland and Germany). Samples will be stored at –80°C until being analysed centrally (Ireland) after completion of the trial. Gut microbiome diversity will be assessed using 16S rRNA sequencing.

#### Muscle motor unit behaviour and muscle signalling

Motor unit behaviour^(82)^ will be determined by high-density electromyography on the vastus lateralis muscle (OT Bioelettronica) at one site (Italy). The primary outcome variables extracted from high-density electromyography will be motor unit mean discharge rate (pps), including discharge rate at recruitment/plateau/de-recruitment, muscle fibre conduction velocity (m/s) and persistent inward currents.

Muscle signalling related to muscle plasticity, metabolism, denervation and capillarisation will also be determined from muscle biopsies, at baseline and post-intervention, from participants who consent at one site (Italy). Biopsies will be taken using MEDAX/BF14100-C0; Bio-Feather with coaxial 14 g 10 cm and subsequently stored at −80°C. Sample analysis will be performed after completion of the trial. The following parameters will then be measured in the sample using western blot with specific antibodies for total proteins or by their activated phosphorylated form: (1) markers of denervation (AChR; agrin/MuSK/Lrp4, NCAM, Myog); (2) markers of protein turnover (atrogin1, MURF1, LC3, BNIP3, Akt-dependent mTOR and FoxO); (3) markers of mitochondrial dynamics (OPA1, DRP1); and (4) markers of energy metabolism pathways (AMPK and PGC1alpha).

#### Acceptability of the trial

To determine acceptability and feasibility of the intervention and overall experience while enrolled in the study, a study-specific questionnaire designed similar to previous studies^(83)^ containing statements (e.g. *I was able to integrate the programme into everyday life*) and five-point Likert scale responses ranging from ‘*Strongly disagree’* to ‘*Strongly agree’* will be administered to participants on completion of the intervention. The questionnaire will also enable participant challenges and suggestions to be reported through open questions (e.g. *What helped you (or made it difficult) to follow through with the programme?*). In addition, for participants in the PD groups, feedback on the PPF products will be recorded at the end of the intervention and during the dietary recall telephone calls with open questions (e.g. *How did you integrate the PPF products into your diet?).*

### Adverse effects and events

Any adverse effects will be recorded as reported by participants during the trial, for example, during fortnightly telephone calls for PD groups, during PA classes or if a participant contacts the research team to report adverse effects or events at any time during the trial.

### Data management and statistical analysis plan

All data will be collected on questionnaires and record sheets in a de-identified manner using a participant ID number and stored securely according to General Data Protection Regulation (2018)^(84)^ at each site, with electronic data double-entry into a central electronic database stored and backed up at one site (Ireland). The trial coordinating centre Principal Investigators will have access to the full dataset across all sites. Study site Principal Investigators will have direct access to the dataset at their own site and will have access to data from other sites by request. Anonymised data will be made available to other researchers on reasonable request after the main trial manuscript is published.

There are no plans for interim study analysis. After completion of the study data collection, data will be checked and cleaned using descriptive statistics and data visualisation. Outcome data will first be analysed according to the intention-to-treat principle, using a random coefficients generalised linear mixed model for primary and secondary end points. The effects of time, the two interventions and their interaction (following the 2 × 2 factorial design of the trial) and sex will be included as fixed effects. Participant ID and study site will be included as random effects. Residuals will be tested for normality and transformation applied where required, or where the structure of the data is inherently non-normal. 95 % CI will be reported where applicable. Per-protocol analyses and mediation analyses will be conducted to examine to what extent compliance and potential mediating mechanisms explain the impact on the primary and secondary end points. For primary pre-planned contrasts, significance will be accepted as *P* < 0·05. Where groups of secondary outcomes are analysed, the significance level will be adjusted by Bonferroni correction according to the number of responses in the analysis. A correlation matrix will be constructed to assess relationships between outcome variables. Missing data will not be imputed in the primary analysis. Intention-to-treat analyses will be based on the *as-observed* population.

### Ethics and dissemination

Ethical approval has been received from the Human Research Ethics Committees at each trial centre and the trial registered on ClinicalTrials.gov (NCT05608707). The study will be reported according to the Consolidated Standards of Reporting Trials (CONSORT) guidelines for parallel-group randomised trials. Participant informed consent will be obtained in writing prior to study enrolment (online Supplemental Appendix 6). Adverse events will be recorded and reported to the relevant ethics committee. Participants are informed they may withdraw with no obligation to provide a reason. If participants choose to provide a reason, the reason will be recorded. Amendments to the trial protocol including changes to study objectives, design, participant population, sample sizes, study procedures or significant administrative aspects are updated on the trial registry and submitted as an amendment for ethical approval at each site.

Trial results will be disseminated through websites, reports, presentations, peer-reviewed journal articles and workshops to a wide audience of stakeholders in the area of healthy ageing including researchers, healthcare providers, policymakers, industry and older adults. Topics and authors proposed for peer-reviewed publication as a journal article will be circulated to the trial principal investigators of the coordinating centres and the consortium principal investigators, with authorship based on established authorship guidelines^(85)^.

## Discussion

The APPETITE trial is the first RCT to evaluate the impact of PD and PA alone or in combination on appetite and related health outcomes in older adults. The PD intervention involves the participant incorporating two PPF powder supplements into the diet and receiving personalised dietary advice to achieve defined daily energy and protein targets. The PA intervention involves supervised resistance exercise classes twice per week and home-based walking three times per week.

Although previous studies have investigated the effects of protein supplementation, alone or with PA on sarcopenia-related outcomes^(86,87)^, nutritional outcomes, particularly appetite, have received little attention. Alongside plant protein supplementation, the inclusion of fibre, identified as lacking in diets of many older adults and appealing to those with poor appetite^(26,27,35,88)^, is a novel aspect of the present study. Furthermore, previous supplementation studies have focused primarily on providing a supplement, without considering usual dietary habits and preferences, thus not following a personalised approach which is recommended by consensus guidelines^(2)^.

The PPF supplements that are provided to participants as part of the PD intervention were developed based on prior research and initial data from earlier work packages as part of the wider APPETITE project^(49)^. The supplements provided consist of a blend of plant proteins to optimise the amino acid content and reduce the limitations associated with providing a single plant protein source^(89)^. The PPF supplements were selected from a range of six blends developed for the trial, initially based on results from sensory testing among older adults to optimise compliance, and subsequently from assessment of peripheral amino acid appearance following consumption.

As noted in the methods section, during the initial months of the trial, the inclusion criteria requiring participants to have a SNAQ score </= 14 was removed. This criterion was adjusted for several reasons including difficulty encountered in recruitment and acknowledging both findings from work package 1 of the wider APPETITE project and limited validation studies of SNAQ as an assessment of appetite *per se*. Work package 1 involved semi-structured interviews with older adults with ‘poor appetite’ as defined by SNAQ (</=14). A key finding was that despite their SNAQ score, many participants did not recognise they had a poor appetite or were reluctant to acknowledge their appetite loss^(90)^. This difficulty in self-identifying appetite loss may likely contribute to challenges in recruiting community-dwelling older adults with a ‘poor appetite’. While the inclusion of specific ‘appetite loss’ criteria may be more feasible in a clinical setting (where appetite loss is more widespread and more pronounced) as part of regular patient screening and appropriate for drug trials, such criteria may be less applicable for lifestyle intervention trials targeting community-dwelling older adults. In addition, reduced appetite and energy intake occurs in healthy older compared with younger adults^(10)^, and is not defined by SNAQ score specifically. Consequently, the APPETITE trial will include older adults not defined by a specific ‘appetite status’ at baseline.

A key strength of the design is the multi-centre design, thereby enhancing the generalisability of findings and potential future implementation of the interventions. Further strengths include the 2 × 2 design, allowing for the impact of PD and PA separately and in combination to be elucidated. In addition, given additional facilities and expertise available at some centres (such as muscle biopsy and analysis), insight into important additional outcomes will be obtained in subsamples. The integration of comprehensive assessment, including body composition, gut microbiome, blood biomarkers, functional, metabolic and health-related outcomes, will give insight into mechanisms contributing to any changes observed.

The time required to improve some of the outcomes assessed is unclear and may require a longer duration than the 12-week intervention in the APPETITE RCT; however, effects of protein supplementation and exercise on appetite have previously been demonstrated in 12-week trials across various populations^(36,91)^.

Due to the nature of the intervention, one limitation is that it is not possible to blind participants to treatment allocation. An additional limitation is the lack of blinding of researchers who are involved in both the administration of the intervention and data collection due to logistical constraints. However, data will be analysed anonymously by a blinded researcher to minimise detection bias.

In summary, the APPETITE multi-centre RCT will provide novel insight on the impact of PD (incorporating PPF products), PA and their combination on appetite, and other nutritional, functional, clinical and metabolic outcomes in community-dwelling older adults. This will advance knowledge on how these interventions influence appetite, dietary intake and nutritional status and contribute to future research in the field. This will ultimately inform guidelines to contribute to a better nutritional status and quality of life for older Europeans.

### Trial status

This protocol is version 4 registered on 29/9/2023. Recruitment began in September 2022 and finished in April 2024, with the last participant completing their final test visit in July 2024.

## Supporting information

Horner et al. supplementary material 1 Horner et al. supplementary material

Horner et al. supplementary material 2 Horner et al. supplementary material

Horner et al. supplementary material 3 Horner et al. supplementary material

Horner et al. supplementary material 4 Horner et al. supplementary material

Horner et al. supplementary material 5 Horner et al. supplementary material

Horner et al. supplementary material 6 Horner et al. supplementary material

## Supplementary material

For supplementary material/s referred to in this article, please visit https://doi.org/10.1017/S0007114524002125

## References

[1] Roberts HC , Lim SER , Cox NJ , et al. (2019) The challenge of managing undernutrition in older people with frailty. Nutrients 11, 808. 30974825 10.3390/nu11040808 PMC6521101

[2] Volkert D , Beck AM , Cederholm T , et al. (2019) ESPEN guideline on clinical nutrition and hydration in geriatrics. Clin Nutr 38, 10–47. 30005900 10.1016/j.clnu.2018.05.024

[3] Morley JE (2017) Anorexia of ageing: a key component in the pathogenesis of both sarcopenia and cachexia. J Cachexia Sarcopenia Muscle 8, 523–526. 28452130 10.1002/jcsm.12192 PMC5566640

[4] Cruz-Jentoft AJ , Baeyens JP , Bauer JM , et al. (2010) Sarcopenia: European consensus on definition and diagnosis. Age Ageing 39, 412–423. 20392703 10.1093/ageing/afq034 PMC2886201

[5] Clegg A , Young J , Iliffe S , et al. (2013) Frailty in elderly people. Lancet 381, 752–762. 23395245 10.1016/S0140-6736(12)62167-9 PMC4098658

[6] Morley JE (2013) Pathophysiology of the anorexia of aging. Curr Opin Clin Nutr Metab Care 16, 27–32. 23041615 10.1097/MCO.0b013e328359efd7

[7] Volkert D , Kiesswetter E , Cederholm T , et al. (2019) Development of a model on determinants of malnutrition in aged persons: a MaNuEL project. Gerontol Geriatr Med 5, 233372141985843. 10.1177/2333721419858438 PMC6589946 31259204

[8] Blundell J , De Graaf C , Hulshof T , et al. (2010) Appetite control: methodological aspects of the evaluation of foods. Obes Rev 11, 251–270. 20122136 10.1111/j.1467-789X.2010.00714.x PMC3609405

[9] Soenen S & Chapman IM (2013) Body weight, anorexia, and undernutrition in older people. J Am Med Dir Assoc 14, 642–648. 23522494 10.1016/j.jamda.2013.02.004

[10] Giezenaar C , Chapman I , Luscombe-Marsh N , et al. (2016) Ageing is associated with decreases in appetite and energy intake—a meta-analysis in healthy adults. Nutrients 8, 28. 26751475 10.3390/nu8010028 PMC4728642

[11] Malafarina V , Uriz-Otano F , Gil-Guerrero L , et al. (2013) The anorexia of ageing: physiopathology, prevalence, associated comorbidity and mortality. A systematic review. Maturitas 74, 293–302. 23415063 10.1016/j.maturitas.2013.01.016

[12] Cox NJ , Morrison L , Ibrahim K , et al. (2020) New horizons in appetite and the anorexia of ageing. Age Ageing 49, 526–534. 32043144 10.1093/ageing/afaa014

[13] Fluitman KS , Hesp AC , Kaihatu RF , et al. (2021) Poor taste and smell are associated with poor appetite, macronutrient intake, and dietary quality but not with undernutrition in older adults. J Nutr 151, 605–614. 33561272 10.1093/jn/nxaa400 PMC7948202

[14] Franceschi C , Capri M , Monti D , et al. (2007) Inflammaging and anti-inflammaging: a systemic perspective on aging and longevity emerged from studies in humans. Mech Ageing Dev 128, 92–105. 17116321 10.1016/j.mad.2006.11.016

[15] de Souto Barreto P , Cesari M , et al. (2022) Appetite loss and anorexia of aging in clinical care: an ICFSR task force report. J Frailty Aging 11, 129–134. 35441188 10.14283/jfa.2022.14 PMC8898654

[16] Cox NJ , Bowyer RCE , Ni Lochlainn M , et al. (2021) The composition of the gut microbiome differs among community dwelling older people with good and poor appetite. J Cachexia Sarcopenia Muscle 12, 368–377. 33580637 10.1002/jcsm.12683 PMC8061352

[17] Fielding RA , Landi F , Smoyer KE , et al. (2023) Association of anorexia/appetite loss with malnutrition and mortality in older populations: a systematic literature review. J Cachexia Sarcopenia Muscle 14, 706–729. 36807868 10.1002/jcsm.13186 PMC10067499

[18] Bauer J , Biolo G , Cederholm T , et al. (2013) Evidence-based recommendations for optimal dietary protein intake in older people: a position paper from the PROT-AGE study group. J Am Med Dir Assoc 14, 542–559. 23867520 10.1016/j.jamda.2013.05.021

[19] Deutz NEP , Bauer JM , Barazzoni R , et al. (2014) Protein intake and exercise for optimal muscle function with aging: recommendations from the ESPEN expert group. Clin Nutr 33, 929–936. 24814383 10.1016/j.clnu.2014.04.007 PMC4208946

[20] Murphy CH , Oikawa SY & Phillips SM (2016) Dietary protein to maintain muscle mass in aging: a case for per-meal protein recommendations. J Frailty Aging 5, 49–58. 26980369 10.14283/jfa.2016.80

[21] Mendonça N , Hengeveld LM , Visser M , et al. (2021) Low protein intake, physical activity, and physical function in European and North American community-dwelling older adults: a pooled analysis of four longitudinal aging cohorts. Am J Clin Nutr 114, 29–41. 33829238 10.1093/ajcn/nqab051 PMC8246618

[22] McKeown NM , Fahey GC , Slavin J , et al. (2022) Fibre intake for optimal health: how can healthcare professionals support people to reach dietary recommendations?. BMJ 378, e054370. 35858693 10.1136/bmj-2020-054370 PMC9298262

[23] Scientific Advisory Committee on Nutrition (SACN) (2021) SACN statement on nutrition and older adults living in the community. https://www.gov.uk/government/publications/sacn-statement-on-nutrition-and-older-adults (accessed July 2024). 10.1017/S0007114520005024 33334383

[24] Scientific Advisory Committee on Nutrition (SACN) (2015) Carbohydrates and Health http://www.gov.uk/government/uploads/system/uploads/attachment_data/file/445503/SACN_Carbohydrates_and_Health.pdf (accessed July 2024).

[25] EFSA Panel on Dietetic Products, Nutrition, and Allergies (NDA) (2010) Scientific opinion on dietary reference values for carbohydrates and dietary fibre. EFSA J 8, 1462.

[26] Kehoe L , Walton J & Flynn A (2019) Nutritional challenges for older adults in Europe: current status and future directions. Proc Nutr Soc 78, 221–233. 30696516 10.1017/S0029665118002744

[27] Van Der Meij BS , Wijnhoven HAH , Lee JS , et al. (2017) Poor appetite and dietary intake in community-dwelling older adults. J Am Geriatr Soc 65, 2190–2197. 28744853 10.1111/jgs.15017 PMC5641246

[28] Linschooten JO , Verwijs MH , Beelen J , et al. (2021) Low awareness of community-dwelling older adults on the importance of dietary protein: new insights from four qualitative studies. J Nutr Sci 10, e102. 35059183 10.1017/jns.2021.92 PMC8727701

[29] Hengeveld LM , Boer JMA , Gaudreau P , et al. (2020) Prevalence of protein intake below recommended in community-dwelling older adults: a meta-analysis across cohorts from the PROMISS consortium. J Cachexia Sarcopenia Muscle 11, 1212–1222. 32548960 10.1002/jcsm.12580 PMC7567142

[30] Morris S , Cater JD , Green MA et al. (2020) Inadequacy of protein intake in older UK adults. Geriatrics 5:6. 32059533 10.3390/geriatrics5010006 PMC7151458

[31] Stephen AM , Champ MMJ , Cloran SJ , et al. (2017) Dietary fibre in Europe: current state of knowledge on definitions, sources, recommendations, intakes and relationships to health. Nutr Res Rev 30, 149–190. 28676135 10.1017/S095442241700004X

[32] Ben-Harchache S , Roche HM , Corish CA , et al. (2021) The impact of protein supplementation on appetite and energy intake in healthy older adults: a systematic review with meta-analysis. Adv Nutr 12, 490–502. 33037427 10.1093/advances/nmaa115 PMC8009738

[33] Clark MJ & Slavin JL (2013) The effect of fiber on satiety and food intake: a systematic review. J Am Coll Nutr 32, 200–211. 23885994 10.1080/07315724.2013.791194

[34] Alyousif Z , Mendoza DR , Auger J , et al. (2020) Gastrointestinal tolerance and microbiome response to snacks fortified with pea hull fiber: a randomized trial in older adults. Curr Dev Nutr 4, nzaa005. 10.1093/cdn/nzaa005 PMC6994441 32025615

[35] Van Der Meij BS , Wijnhoven HAH , Finlayson GS , et al. (2015) Specific food preferences of older adults with a poor appetite. A forced-choice test conducted in various care settings. Appetite 90, 168–175. 25772198 10.1016/j.appet.2015.03.011

[36] Johnson KO , Holliday A , Mistry N , et al. (2021) An increase in fat-free mass is associated with higher appetite and energy intake in older adults: a randomised control trial. Nutrients 13, 141. 33401473 10.3390/nu13010141 PMC7824356

[37] So D , Whelan K , Rossi M , et al. (2018) Dietary fiber intervention on gut microbiota composition in healthy adults: a systematic review and meta-analysis. Am J Clin Nutr 2018(107), 965–983. 10.1093/ajcn/nqy041 29757343

[38] Cummings JH , Antoine JM , Azpiroz F , et al. (2004) PASSCLAIM-gut health and immunity. Eur J Nutr 43, 118–173. 10.1007/s00394-004-1205-4 15221356

[39] Fluitman KS , Davids M , Olofsson LE , et al. (2022) Gut microbial characteristics in poor appetite and undernutrition: a cohort of older adults and microbiota transfer in germ-free mice. J Cachexia Sarcopenia Muscle 13, 2188–2201. 35698917 10.1002/jcsm.13002 PMC9397553

[40] Barger K , Langsetmo L , Orwoll ES , et al. (2020) Investigation of the diet-gut-muscle axis in the osteoporotic fractures in men study. J Nutr Health Aging 24, 445–452. 32242213 10.1007/s12603-020-1344-1 PMC7524010

[41] Prokopidis K , Chambers E , Ni Lochlainn M , et al. (2021) Mechanisms linking the gut-muscle axis with muscle protein metabolism and anabolic resistance: implications for older adults at risk of sarcopenia. Front Physiol 12, 770455. 10.3389/fphys.2021.770455 PMC8576575 34764887

[42] Messina M , Lynch H , Dickinson JM , et al. (2018) No difference between the effects of supplementing with soy protein versus animal protein on gains in muscle mass and strength in response to resistance exercise. Int J Sport Nutr Exerc Metab 28, 674–685. 29722584 10.1123/ijsnem.2018-0071

[43] Martin A , Ecklu-Mensah G , Ha CWY , et al. (2021) Gut microbiota mediate the FGF21 adaptive stress response to chronic dietary protein-restriction in mice. Nat Commun 12, 3838. 34158480 10.1038/s41467-021-24074-z PMC8219803

[44] Grasso AC , Hung Y , Olthof MR , et al. (2019) Older consumers’ readiness to accept alternative, more sustainable protein sources in the European Union. Nutrients 11, 1904. 31443177 10.3390/nu11081904 PMC6723411

[45] Pimentel D & Pimentel M (2003) Sustainability of meat-based and plant-based diets and the environment. Am J Clin Nutr 78, 660–663. 10.1093/ajcn/78.3.660S 12936963

[46] Kirwan RP , Mazidi M , Rodríguez García C , et al. (2022) Protein interventions augment the effect of resistance exercise on appendicular lean mass and handgrip strength in older adults: a systematic review and meta-analysis of randomized controlled trials. Am J Clin Nutr 115, 897–913. 34673936 10.1093/ajcn/nqab355

[47] Clegg ME & Godfrey A (2018) The relationship between physical activity, appetite and energy intake in older adults: a systematic review. Appetite 128, 145–151. 29885385 10.1016/j.appet.2018.05.139

[48] Crabtree DR , Cox NJ , Lim SER , et al. (2023) Enhancing the management of anorexia of ageing to counteract malnutrition: are physical activity guidelines optimal?. Aging Clin Exp Res 35, 427–431. 36662481 10.1007/s40520-022-02317-3 PMC9894952

[49] Volkert D , Corish CA , Dardevet D , et al. (2021) Innovative plAnt protein fibre and physical activity solutions to address poor appEtite and prevenT undernutrITion in oldEr adults – APPETITE. Nutr Bull 46, 486–496. 10.1017/S0007114524002125 PMC11557289 39387205

[50] Chan AW , Tetzlaff JM , Gotzsche PC , et al. (2013) SPIRIT 2013 explanation and elaboration: guidance for protocols of clinical trials. BMJ 346, e7586–e7586. 23303884 10.1136/bmj.e7586 PMC3541470

[51] Gregersen NT , Flint A , Bitz C , et al. (2008) Reproducibility and power of ad libitum energy intake assessed by repeated single meals. Am J Clin Nutr 87, 1277–1281. 18469250 10.1093/ajcn/87.5.1277

[52] Wijnhoven HAh , Van Der Meij BS & Visser M (2015) Variety within a cooked meal increases meal energy intake in older women with a poor appetite. Appetite 95, 571–576. 26321418 10.1016/j.appet.2015.08.029

[53] Fergusson D (2002) Post-randomisation exclusions: the intention to treat principle and excluding patients from analysis. BMJ 325, 652–654. 12242181 10.1136/bmj.325.7365.652 PMC1124168

[54] Bernabei R , Landi F , Calvani R , et al. (2022) Multicomponent intervention to prevent mobility disability in frail older adults: randomised controlled trial (SPRINTT project). BMJ 11, e068788. 10.1136/bmj-2021-068788 PMC9092831 35545258

[55] Reinders I , Visser M , Jyväkorpi SK , et al. (2022) The cost effectiveness of personalized dietary advice to increase protein intake in older adults with lower habitual protein intake: a randomized controlled trial. Eur J Nutr 61, 505–520. 34609621 10.1007/s00394-021-02675-0 PMC8490609

[56] De Marco Castro E , Valli G , Buffière C , et al. (2022) Peripheral amino acid appearance is lower following plant protein fibre products, compared to whey protein and fibre ingestion, in healthy older adults despite optimised amino acid profile. Nutrients 15, 35. 36615694 10.3390/nu15010035 PMC9824653

[57] HSE (2017) Nutrition Supports. https://www.hse.ie/eng/services/list/2/primarycare/community-funded-schemes/nutrition-supports/ (accessed January 2024).

[58] FAO (2013) Dietary protein quality evaluation in human nutrition. Report of an FAQ expert consultation. FAO Food Nutr Pap 92, 1–66. 26369006

[59] Regan E , O’Neill GJ , Hutchings SC , et al. (2019) Exploring how age influences sensory perception, thirst and hunger during the consumption of oral nutritional supplements using the check-all-that-apply methodology. Food Qual Prefer 78, 103736.

[60] Pahor M , Guralnik JM , Ambrosius WT , et al. (2014) Effect of structured physical activity on prevention of major mobility disability in older adults: the LIFE study randomized clinical trial. JAMA 311, 2387. 24866862 10.1001/jama.2014.5616 PMC4266388

[61] Hurst C , Robinson SM , Witham MD , et al. (2022) Resistance exercise as a treatment for sarcopenia: prescription and delivery. Age Ageing 51, afac003. 10.1093/ageing/afac003 PMC8840798 35150587

[62] Borg G (1970) Perceived exertion as an indicator of somatic stress. Scand J Rehabil Med 2, 92–98. 5523831

[63] Bauer JM , Verlaan S , Bautmans I , et al. (2015) Effects of a vitamin D and leucine-enriched whey protein nutritional supplement on measures of sarcopenia in older adults, the PROVIDE study: a randomized, double-blind, placebo-controlled trial. J Am Med Dir Assoc 16, 740–747. 26170041 10.1016/j.jamda.2015.05.021

[64] Gibbons C , Finlayson G , Dalton M , et al. (2014) Metabolic phenotyping guidelines: Studying eating behaviour in humans. J Endocrinol 222, G1–12. 25052364 10.1530/JOE-14-0020

[65] Turicchi J , O’Driscoll R , Finlayson G , et al. (2020) Associations between the proportion of fat-free mass loss during weight loss, changes in appetite, and subsequent weight change: results from a randomized 2-stage dietary intervention trial. Am J Clin Nutr 111, 536–544. 31950141 10.1093/ajcn/nqz331

[66] Flint A , Raben A , Blundell J , et al. (2000) Reproducibility, power and validity of visual analogue scales in assessment of appetite sensations in single test meal studies. Int J Obes 24, 38–48. 10.1038/sj.ijo.0801083 10702749

[67] Parker BA , Sturm K , MacIntosh CG , et al. (2004) Relation between food intake and visual analogue scale ratings of appetite and other sensations in healthy older and young subjects. Eur J Clin Nutr 58, 212–218. 14749739 10.1038/sj.ejcn.1601768

[68] Parker BA , Ludher AK , Khai Loon T , et al. (2004) Relationships of ratings of appetite to food intake in healthy older men and women. Appetite 43, 227–233. 15527924 10.1016/j.appet.2004.05.004

[69] Kyle UG , Genton L , Karsegard L , et al. (2001) Single prediction equation for bioelectrical impedance analysis in adults aged 20–94 years. Nutr 17, 248–253. 10.1016/s0899-9007(00)00553-0 11312069

[70] Sergi G , De Rui M , Veronese N , et al. (2015) Assessing appendicular skeletal muscle mass with bioelectrical impedance analysis in free-living Caucasian older adults. Clin Nutr 34, 667–673. 25103151 10.1016/j.clnu.2014.07.010

[71] Wilson MMG , Thomas DR , Rubenstein LZ , et al. (2005) Appetite assessment: simple appetite questionnaire predicts weight loss in community-dwelling adults and nursing home residents. Am J Clin Nutr 82, 1074–1081. 16280441 10.1093/ajcn/82.5.1074

[72] Mathey MF (2001) Assessing appetite in dutch elderly with the appetite, hunger and sensory perception (AHSP) questionnaire. J Nutr Health Aging 5, 22–28. 11250665

[73] Nutritics (2024) Nutritics. Dublin. Available from: https://www.nutritics.com (accessed January 2024).

[74] Gnagnarella P , Salvini S & Parpinel M (2024) Food Composition Database for Epidemiological Studies in Italy (Banca Dati di Composizione degli Alimenti per Studi Epidemiologici in Italia – BDA). Italy: BDA.

[75] Moffit RE , Qiao YS , Moored KD , et al. (2021) Estimating cardiorespiratory fitness in older adults using a usual-paced 400-m long-distance corridor walk. J Am Geriatr Soc 69, 3328–3330. 34269423 10.1111/jgs.17360 PMC8595496

[76] Guralnik JM , Simonsick EM , Ferrucci L , et al. (1994) A short physical performance battery assessing lower extremity function: association with self-reported disability and prediction of mortality and nursing home admission. J Gerontol 49, M85–94. 8126356 10.1093/geronj/49.2.m85

[77] Washburn RA , Smith KW , Jette AM , et al. (1993) The physical activity scale for the elderly (PASE): development and evaluation. J Clin Epidemiol 46, 153–162. 8437031 10.1016/0895-4356(93)90053-4

[78] Folstein MF , Folstein SE & McHugh PR (1975) Mini-mental state. J Psychiatr Res 12, 189–198. 1202204 10.1016/0022-3956(75)90026-6

[79] Stunkard AJ & Messick S (1985) The three-factor eating questionnaire to measure dietary restraint, disinhibition and hunger. J Psychosom Res 29, 71–83. 3981480 10.1016/0022-3999(85)90010-8

[80] Radloff LS (1977) The CES-D scale: a self-report depression scale for research in the general population. Appl Psychol Meas 1, 385–401.

[81] Herdman M , Gudex C , Lloyd A , et al. (2011) Development and preliminary testing of the new five-level version of EQ-5D (EQ-5D-5L). Qual Life Res 20, 1727–1736. 21479777 10.1007/s11136-011-9903-x PMC3220807

[82] Valli G , Sarto F , Casolo A , et al. (2024) Lower limb suspension induces threshold-specific alterations of motor units properties that are reversed by active recovery. J Sport Health Sci 13, 264–276. 37331508 10.1016/j.jshs.2023.06.004 PMC10980901

[83] Niskanen RT , Reinders I , Wijnhoven HAH , et al. (2023) The feasibility of a 6-month dietary intervention aiming to increase protein intake among community-dwelling older adults with low habitual protein intake: a secondary analysis of the PROMISS randomised controlled trial. J Hum Nutr Diet 36, 1811–1820. 37347495 10.1111/jhn.13197

[84] Regulations (2016) Regulation (EU) 2016/679 of the European parliament and of the council of 27 April 2016 on the protection of natural persons with regard to the processing of personal data and on the free movement of such data, and repealing directive 95/46/EC (general data protection regulation). J Eur Union L 119:1–88.

[85] International Committee of Medical Journal Editors (2024) Defining the roles of authors and contributors. https://www.icmje.org/recommendations/browse/roles-and-responsibilities/defining-the-role-of-authors-and-contributors.html (accessed April 2024).

[86] Hou L , Lei Y , Li X , et al. (2019) Effect of protein supplementation combined with resistance training on muscle mass, strength and function in the elderly: a systematic review and meta-analysis. J Nutr Health Aging 23, 451–458.31021362 10.1007/s12603-019-1181-2

[87] ten Haaf DSM , Nuijten MAH , Maessen MFH , et al. (2018) Effects of protein supplementation on lean body mass, muscle strength, and physical performance in nonfrail community-dwelling older adults: a systematic review and meta-analysis. Am J Clin Nutr 108, 1043–1059. 30475963 10.1093/ajcn/nqy192

[88] Ter Borg S , Verlaan S , Mijnarends DM , et al. (2015) Macronutrient intake and inadequacies of community-dwelling older adults, a systematic review. Ann Nutr Metab 66, 242–255. 26183836 10.1159/000435862

[89] Pinckaers PJM , Trommelen J , Snijders T , et al. (2021) The anabolic response to plant-based protein ingestion. Sports Med 51, 59–74. 34515966 10.1007/s40279-021-01540-8 PMC8566416

[90] Dismore L , Sayer A & Robinson S (2024) Exploring the experience of appetite loss in older age: insights from a qualitative study. BMC Geriatr 24, 117. 38297212 10.1186/s12877-024-04732-9 PMC10829396

[91] King NA , Caudwell PP , Hopkins M , et al. (2009) Dual-process action of exercise on appetite control: increase in orexigenic drive but improvement in meal-induced satiety. Am J Clin Nutr 90, 921–927. 19675105 10.3945/ajcn.2009.27706

