# Supplementary material for: Plant protein, fibre and physical activity solutions to address poor appetite and prevent undernutrition in older adults: study protocol for the APPETITE randomised controlled trial
Source: Br J Nutr. 2024 Oct 10;132(6):823–34. doi: 10.1017/S0007114524002125 (PMC11557289; doi:10.1017/S0007114524002125)
Supplement: Horner et al. supplementary material 2 — Horner et al. supplementary material [file S0007114524002125sup002.pdf]

Appendix 2 Composition of the coordinating centre, study-site principal investigators, co- investigators and scientific advisory board roles

Table S1 Coordinating Centre, Study-Site Principal Investigators and External Advisory Board Members

**Name**	**Affiliation**	**Role**
Prof Clare Corish	University College Dublin, Ireland	Dependent - Coordinating Centre, Trial Principal Investigator
Dr Katy Horner	University College Dublin, Ireland	Dependent - Coordinating Centre, Trial Principal Investigator
Prof Helen Roche	University College Dublin, Ireland	Dependent - Coordinating Centre, Trial Principal Investigator
Prof Dorothee Volkert	Friedrich-Alexander-Universitat (FAU), Germany	Dependent - Study Site Principal Investigator, APPETITE Consortium Lead Principal Investigator
Prof Giuseppe De Vito	University of Padua, Italy	Dependent - Study Site Principal Investigator
Prof Marjolein Visser	Vrije Universiteit (VU) Amsterdam, The Netherlands	Dependent – Trial Principal Investigator – Scientific Advisor
Dr Dominique Dardevet	Institut National De Recheche Pour L'Agriculture, L'Alimentation et L'Environment (INRAE), France	Dependent – Co- Investigator
Dr Christelle Guillet	Institut National De Recheche Pour L'Agriculture, L'Alimentation et L'Environment (INRAE), France	Dependent – Co-Investigator
Prof Harriet Jager-Wittenar	Hanze University of Applied Sciences, The Netherlands	Independent – External Advisory Board, Scientific Advisor
Prof Ellen Freiberger	Friedrich-Alexander-Universitat (FAU), Germany	Independent – External Advisory Board, Scientific Advisor
Prof Hannelore Daniel	Technical University of Munich, Germany	Independent – External Advisory Board, Scientific Advisor
Prof Claudio Franceschi	University of Bologna, Italy	Independent – External Advisory Board, Scientific Advisor
Prof Alfonso Cruz-Jentoft	European University of Madrid, Spain	Independent – External Advisory Board, Scientific Advisor
Prof Tommy Cederholm	Uppsala University, Sweden	Independent – External Advisory Board, Scientific Advisor
