# Supplementary material for: Plant protein, fibre and physical activity solutions to address poor appetite and prevent undernutrition in older adults: study protocol for the APPETITE randomised controlled trial
Source: Br J Nutr. 2024 Oct 10;132(6):823–34. doi: 10.1017/S0007114524002125 (PMC11557289; doi:10.1017/S0007114524002125)
Supplement: Horner et al. supplementary material 3 — Horner et al. supplementary material [file S0007114524002125sup003.pdf]

APPENDIX 3. Medical Conditions and Medications for Exclusion

List of medical conditions (as self-reported by participant based on previous medical doctor diagnosis, unless otherwise stated).

**Condition**
**Active inflammatory bowel disease, active Crohn's disease, ulcerative colitis, coeliac disease, chronic pancreatitis or other disorder potentially causing malabsorption**
**Alzheimer´s disease**
**Severe arthritis, such as awaiting joint replacement**, or that would interfere with the ability to participate fully in either study arm
**Bariatric surgery previously or intention to undergo bariatric surgery** within the next 12 months
**Cancer** - active malignant cancer or history of malignancy **within the last 2 years** (with exception of local basal and squamous cell skin cancer).
**Cardiovascular disease** – including current unstable angina; myocardial infarction; coronary revascularization procedures; stroke (either ischemic or haemorrhagic, including transient ischemic attacks); symptomatic peripheral artery disease that required surgery or was diagnosed with vascular imaging techniques; ventricular arrhythmia; **uncontrolled** atrial fibrillation; congestive heart failure Class III or IV (New York Heart Association); hypertrophic myocardiopathy; and history of aortic aneurism ≥5.5 cm in diameter or aortic aneurism surgery<br>**- within the past six months,**<br>**- uncontrolled/unstable conditions**<br>**- as diagnosed by a medical doctor.**
**Chronic inflammatory diseases (note some are also listed elsewhere as exclusion):**<br>**Organ-specific chronic inflammatory disorders including:**<br>o 'Severe' psoriasis and similar disorders (excluding pityriasis rosea and related disorders);<br>o Bullous skin diseases;<br>o Asthma **(If newly diagnosed or poorly controlled)**<br>o Ankylosing spondylitis.<br>o Liver disease e.g. cirrhosis (fatty liver disease allowed), as diagnosed by a medical doctor.<br><br>**Systemic autoimmune disorders:** e.g. / including<br>o Systemic lupus,<br>o Erythematosus,<br>o Scleroderma,<br>o Sjogren syndrome as well as other diffuse connective tissue disorders<br>o Systemic vasculitis, including polyarteritis nodosa, Wegener's granulomatosis, giant cell arteritis, and related conditions.
**Chronic kidney disease requiring dialysis**
**COPD, or other lung disease** requiring regular use of corticosteroids or of supplemental oxygen
**Diabetes mellitus (type 1 and 2)** (History of gestational diabetes mellitus is allowed)
**Hip fracture, hip or knee replacement, or spinal surgery** in the past 6 months
**History of extensive small or large bowel resection**
**Neurological disorders** including MS, Parkinsons disease, cerebral palsy

**Psychiatric disorder current diagnosis: schizophrenia, bipolar disease, eating disorders, depression or other psychiatric disorder** within the last 6 months.
**Systolic blood pressure above 200 mmHg** and/or **diastolic blood pressure above 110 mmHg** whether on or off treatment for hypertension. If being treated, stable treatment (i.e. no change in treatment, either dose, type of medication or other changes) within last 3 months is required.
**Transmissible blood-borne diseases e.g. hepatitis B, HIV**
**Any other condition** that is **judged by the investigator** may interfere with the adherence to the study protocol
Other illness of such severity that **life expectancy** is less than 12 months
**Temporary:**<br>**Acute** disease (not listed above) should be excluded, until participant has fully recovered.<br>**Awaiting surgical procedure** that may interfere with the study protocol.

Note: Dysphagia/dental/oral problems are not direct exclusion criteria. However, if severe enough, participants with these conditions will be ruled out in screening as participants may identify they are not able to consume study test foods.

List of medications for exclusion

Medication Excluded	Comments/Duration of medication intake:
o Weight loss medications including orlistat, liraglutide, semaglutide (either prescription, over the counter or herbal)	Current use or use within past 6 months
o Growth hormone, estrogens, progesterone (megestrol etc), or testosterone	Current use or use or use in past 3 months
o Corticosteroids (excluding inhaled and topical steroids)	Current use or use within past month.
o Psychoactive medication,	Current use or use within past month
o Epileptic medication,	Current use or use within past month
o Oral antidiabetics, insulin	Current use or use within past month
o Anti-biotics, antivirals	Current use or use within past month
o Anti-arrthymics, anti-hypertensives, thyroid, statins, diuretics, heart failure medication, bronchodilators	Exclude if prescription has changed during last 3 months. If on stable prescription participant can be included.
o Anti-inflammatories (e.g. aspirin, ibuprofen etc)	**Chronic prescription:** Exclude if prescription has changed during last 3 months. If on stable prescription participant can be included. **If acute use (e.g. for headache/fever etc):** appetite testing should not be performed within one week of taking acutely. Reschedule for later if required.
o Anti-fungals, muscle relaxants, migraine medications	**Chronic prescription (e.g. for nail/blood/lung infection, chronic pain etc):** Exclude if prescription has changed during last 3 months. If on stable prescription can be included. **Acute use (e.g. for skin infection, muscle spasm, acute migraine etc):** appetite testing should not be performed within one week of taking acutely. Reschedule for later if required.
o Anti-depressants, antipsychotics, mood stabilisers, hypnotics	Exclude if prescription has changed during last 3 months. Should be excluded by medical condition if reporting depression, psychotic disorder or similar diagnosis.
