# Supplementary material for: Plant protein, fibre and physical activity solutions to address poor appetite and prevent undernutrition in older adults: study protocol for the APPETITE randomised controlled trial
Source: Br J Nutr. 2024 Oct 10;132(6):823–34. doi: 10.1017/S0007114524002125 (PMC11557289; doi:10.1017/S0007114524002125)
Supplement: Horner et al. supplementary material 4 — Horner et al. supplementary material [file S0007114524002125sup004.pdf]

Appendix 4. Nutritional Values of PPF products*

**Value (unit)**		**Product**	
		**PPF A**	**PPF B**
Energy	kJ/100g	1805	1741
Energy	kcal/100g	429	413
Protein	g/100g	61.2	68.4
Carbohydrate	g/100g	21.1	16.7
Sugar	g/100g	<2.5	<2.5
Fructose	g/100g	<0.5	<0.5
Glucose	g/100g	<0.5	<0.5
Lactose	g/100g	<0.5	<0.5
Maltose	g/100g	<0.5	<0.5
Sucrose	g/100g	1.05	<0.5
Fat	g/100g	7.9	4.8
Of which saturated fatty acids	g/100g	1.5	1.3
Of which monounsaturated fatty acids	g/100g	2.7	1.1
Of which polyunsaturated fatty acids	g/100g	3.7	2.3
Thereof trans fatty acids	g/100g	0.00	0.02
Total Dietary Fibre	g/100g	14.2	14.4
Insoluble dietary fibre	g/100g	13.6	12.7
Soluble dietary fibre	g/100g	<1.0	1.7
Water	g/100g	6.2	6.1
Sodium	mg/100g	478	795

PPF, plant protein fibre
*25g of the PPF product is provided in a foil sachet. The foil sachet is labelled with 'PPF A' or 'PPF B' and the best before date, with no other print.
