# Supplementary material for: Plant protein, fibre and physical activity solutions to address poor appetite and prevent undernutrition in older adults: study protocol for the APPETITE randomised controlled trial
Source: Br J Nutr. 2024 Oct 10;132(6):823–34. doi: 10.1017/S0007114524002125 (PMC11557289; doi:10.1017/S0007114524002125)
Supplement: Horner et al. supplementary material 5 — Horner et al. supplementary material [file S0007114524002125sup005.pdf]

Appendix 5. Schedule of Enrolment, Interventions and Assessments

	Enrolment	Baseline Assessments	Post-Allocation (Intervention period)				Close-out
**TIMEPOINT****	*-t₁*		*Week 1*	*Week 4*	*Week 8*	*Week 12/13*	*Week 13*
**ENROLMENT:**							
Eligibility screen[1]	X						
Informed consent	X						
**Random Allocation**	X						
**INTERVENTIONS:**							
Personalised Diet (PD)			———	———	———	———	
Physical Activity (PA)			———	———	———	———	
PD + PA			———	———	———	———	
Control			———	———	———	———	
**ASSESSMENTS:**							
VAS subjective appetite ratings		X					X
Ad libitum test meal energy intake		X					X
VAS test meal palatability ratings		X					X
Sociodemographic data		X					
Self-efficacy in adhering to intervention		X					
Anthropometry (weight, height, calf circ.)		X					X
SNAQ score	X						X
Cardiorespiratory fitness (400 m walk)		X					X
SPPB score		X					X
Handgrip strength		X					X
Leg strength		X					X
Perceived QoL		X					X
Body composition (BIA, BodPod, DXA)		X					X
Resting energy expenditure		X					X
Physical activity (PASE)		X					X
Physical activity (accelerometry)		X				X	
Energy, protein & fibre intake (3-day record)		X				X	
24-hour dietary recall (PD groups only)				X	X		
Cognitive status (MMSE)	X						X
Cognitive dietary restraint (TFEQ)		X					X
Depressive symptoms (CES-D)	X						X
Appetite-related gut peptides		X					X
Plasma metabolomic profiles		X					X
Gut microbiome diversity		X					X

Motor unit signalling/number		X					X
Muscle signalling		X					X
Compliance to intervention							X
Acceptability of intervention							X

BIA, bioelectrical impedance analysis; DXA, dual x-ray absorptiometry; CES-D, Centre of Epidemiology Study – Depression (CES-D) questionnaire; MMSE, Mini Mental State Examination; PASE, Physical Activity Scale for the Elderly; SNAQ, Simplified Nutritional Appetite Questionnaire; SPPB, Short Physical Performance Battery; TFEQ, Three Factor Eating Questionnaire; VAS, visual analogue scales.

[1]Eligibility screen includes telephone screening and in-person assessment.
