# Supplementary material for: Plant protein, fibre and physical activity solutions to address poor appetite and prevent undernutrition in older adults: study protocol for the APPETITE randomised controlled trial
Source: Br J Nutr. 2024 Oct 10;132(6):823–34. doi: 10.1017/S0007114524002125 (PMC11557289; doi:10.1017/S0007114524002125)
Supplement: Horner et al. supplementary material 6 — Horner et al. supplementary material [file S0007114524002125sup006.pdf]

Appendix 6. Consent Form

Effects of innovative plant protein fibre and physical activity on appetite and incidence of undernutrition in older adults.

Participant consent form

Participant Name: ______________________________

Mark box

1. I understand that my participation is voluntary (it is my choice) and that I am free to withdraw from the research at any time without disadvantage, and I will not be compensated for any time I have given. ☐
2. I understand that this study is part of a research project designed to promote scientific knowledge, which has been approved by the Human Ethics Committee and may be of no direct benefit to me personally. The Ethics Committee may wish to inspect the data collected at any time as part of its monitoring activities. ☐
3. I agree that I am not allergic to any of the foods that will be used in this study. ☐
4. I understand the risks associated with COVID-19, and that researchers will follow all protocols (PPE, disinfection, cleaning and food preparation protocols) required to minimize this risk. ☐
5. I agree that findings from the information I provide can be used in scientific reports, publications and presentations at conferences. ☐
6. I understand that all the information I provide will be processed with strict confidentiality, and that my name will not be displayed or identified in any of the outputs from this study. ☐
7. I understand that the data collected as part of this study will be anonymized and will be stored for 10 years. ☐
8. I agree that the stored data may be used for further research questions. ☐
9. I agree that my anonymized data may be shared with other researchers who are working as part of the larger European project team conducting this research. ☐
10. I understand that I do not have to answer any questions in the questionnaires that may make me feel uncomfortable. ☐
11. I agree to have blood samples collected from me, as part of this study, ☐
12. I hereby fully and freely consent to participate in the study, which has fully been explained to me. ☐

....	....	....
Name of participant (in block letters)	Date	Signature
....	....	....
Name of Person taking consent (if different from researcher)	Date	Signature
....	....	....
Researcher	Date	Signature
